# A protocol for the subcellular fractionation of *Saccharomyces cerevisiae* using nitrogen cavitation and density gradient centrifugation

**DOI:** 10.1002/yea.3002

**Published:** 2014-02-20

**Authors:** Yuchong Wang, Kathryn S Lilley, Stephen G Oliver

**Affiliations:** Cambridge Systems Biology Centre and Department of Biochemistry, University of CambridgeUK

**Keywords:** *Saccharomyces*, subcellular fractionation, organelles, nitrogen cavitation, density-gradient centrifugation

## Abstract

Most protocols for yeast subcellular fractionation involve the use of mechanical shear forces to lyse the spheroplasts produced by the enzymatic digestion of the *Saccharomyces cerevisiae* cell wall. These mechanical homogenization procedures often involve the manual use of devices such as the Dounce homogenizer, and so are very operator-dependent and, in consequence, lack reproducibility. Here, we report a highly reproducible method of homogenizing yeast cells based on nitrogen cavitation. This has been optimized to allow efficient release of subcellular compartments that show a high degree of integrity. The protocol remains effective and reproducible across a range of sample volumes and buffer environments. The subsequent separation method, which employs both sucrose and iodixanol density gradients, has been developed to resolve the major membrane-bound compartments of *S. cerevisiae*. We present an integrated protocol that is fast, facile, robust and efficient and that will enable ‘omics’ studies of the subcellular compartments of *S. cerevisiae* and other yeasts.

## Introduction

One of the major differences between eukaryotic cells and those of bacteria and archaea is that eukaryotic cells are subdivided into a number of membrane-bound compartments. This compartmentation has two purposes: first, it increases the total membrane area available for certain classes of chemical reaction (notably electron transfer and oxidative phosphorylation) and thus mitigates the unavoidable reduction in the surface area: volume ratio due to an increase in cell size; second, it allows the separation of related biochemical pathways for the purposes of either regulation (e.g. the separation of transcription in the nucleus from translation in the cytoplasm) or resource management (e.g. the separation of precursor pools for arginine and uridine biosynthesis). Thus, it is essential that the compartmentation of the eukaryotic cell is accurately represented in systems biology models, e.g. in constraint-based or stoichiometric models of the cellular metabolic network (Herrgard *et al*., 2006). In accordance with this, Yuan *et al*. (2013) have demonstrated that the subcellular compartmentation of intracellular reactions significantly improves the fraction of correct predictions for flux distribution in plant metabolism when using a stoichiometric model of the metabolic network.

While the Yeast 4.0 stoichiometric model of the *Saccharomyces cerevisiae* metabolic network (Radrich *et al*., 2010) defines 16 subcellular compartments and comprises all known metabolic reactions represented in the literature, flux balance analysis (FBA) by the compartmented model was less successful than when the decompartmentalized version of the model was used (Pir *et al*., 2010). This suggests that our current view of the compartmentation of the *S. cerevisiae* cell may be inaccurate or incomplete. Therefore, quantitative data on the subcellular distributions of yeast proteins and metabolites would be likely to provide a valuable resource for future work on yeast systems biology.

Acquisition of the data required for such a resource involves performing proteomic and metabolomic analyses on subcellular fractions of yeast. The critical steps of subcellular fractionation include: (a) cell growth under appropriate conditions; (b) preparation of the cell lysate with minimal damage to organelle integrity; (c) an organelle separation protocol that separates the subcellular components of interest into distinct fractions; and (d) accurate identification and quantitative characterization of the membranes/organelles in the resulting fractions (Zinser and Daum, 1995). Successful preparation of subcellular organelles, their isolation and purification requires attention to conditions that may alter the properties and integrity of both cellular organelles and their constituent molecules.

Numerous protocols have been developed for isolating the subcellular compartments of *S. cerevisiae* for use in subsequent studies, e.g. electron microscopy of nuclear core particles (Kiseleva *et al*., 2007) and mitochondrial autophagy (Kanki *et al*., 2011). While there are well-established methods for the preparation of mitochondria, cell wall and nuclear fractions from yeast cell lysates, the separation of major endomembrane organelles is problematic (Gardarin *et al*., 2010; Papanikou and Glick, 2009). In all cases, the small cell size and tough cell wall of *S. cerevisiae* present major challenges to effective and biologically meaningful separation of subcellular compartments.

Cell disruption by nitrogen decompression from a pressurized vessel has been shown to be a rapid and effective way to homogenize mammalian and plant cells (Simpson, 2010). Compared to ultrasonic or mechanical homogenization methods, which may induce protein aggregation (Papanayotou *et al*., 2010), the chemical and physical stresses imposed by nitrogen cavitation on enzymes and subcellular compartments are much less. However, this method is less effective with yeasts, fungi, spores or other cell types with tough cell walls.

Here, we describe a protocol that has a number of advantages over the generally practised Dounce (glass-to-glass) homogenization for the subcellular fractionation of *S. cerevisiae*. We have minimized the potential disruption of the intracellular organelles and critical protein–membrane interactions by combining spheroplast generation and nitrogen cavitation homogenization. The resolution of organellar fractionation has been enhanced by performing both single- and two-step density gradients. This protocol can be adjusted to enhance the separation and recovery of specific organelles or membrane types and should permit the comprehensive proteomic or lipidomic analysis of the subcellular compartments of this yeast.

## Materials and methods

### Cell culture

*Saccharomyces cerevisiae* strain BY4743-Y23925 (*MAT***a**/*MATα his3*Δ*1*/*his3*Δ*1 leu2*Δ*0*/*leu2*Δ*0 ho*::kanMX/*ho*::kanMX) was streaked on YPD (2% peptone, 1% yeast extract, 2% glucose) agar and incubated at 30°C for 24 h. Single colonies were picked to inoculate a preculture (10 ml). An aliquot (200 µl) of the stationary phase preculture was then inoculated into 300 ml YPD medium. The culture was shaken at 250 rpm at 30°C.

### Spheroplast preparation

Mid-exponential phase cells were harvested from YPD cultures by centrifugation at 3000 × *g*. The procedure was adapted from a previous protocol (Rieder and Emr, 2001). Briefly, cells were resuspended and incubated in 100 mm Tris–sulphate buffer (pH 9.4) supplemented with 10 mm dithiothreitol to break the disulphide bonds. The treated cells were digested with Zymolyase 100 T (*β*-1,3-glucan laminaripentaohydrolase; Seikagaku) at a concentration of 5 µg/OD_600_ unit of cells, at 30°C with shaking at 250 rpm. The spheroplasts were harvested by centrifugation at 1500 × *g* at room temperature and washed twice in homogenization medium (HM), consisting of 250 mm sucrose, 10 mm HEPES (pH 7.4), 1 mm EDTA (pH 8.0) and 1 mm DTT, supplemented with complete protease inhibitor mixture (Roche).

### Lysis of spheroplasts using nitrogen cavitation

The spheroplast suspension was transferred into a prechilled cell disruption bomb (Parr 4635, Parr Instrument Co.), which was connected to a nitrogen source. The bomb was then charged and equilibrated at 500 psi on ice for 10 min. Then the pressure was lowered to 350 psi by releasing nitrogen from the inlet valve. After another 10 min of equilibration, the cell homogenate was collected from the outlet valve and cleared of unbroken cells, partially disrupted cells and aggregates by centrifugation at 1000 × *g* at 4°C. A Dounce homogenization method was optimized, using microscopic examination to assess the generated homogenate for spheroplast breakage efficiency, and western blotting to analyse a preliminary density gradient for organelle integrity. The spheroplast suspension in ice-cold homogenization medium was transferred to a prechilled Dounce homogenizer and disrupted by 10 up-and-down strokes of a tight-fitting pestle. The lysate was cleared by withdrawing the supernatant of a 300 × *g* centrifugation at 4°C in order to remove unbroken cells, partially disrupted cells and aggregates.

### Electron microscopy of spheroplast preparations and homogenates

#### Scanning electron microscopy (SEM)

Spheroplasts or cell lysates were washed twice in imidazole hydrochloride buffer and fixed by resuspension in the same buffer containing 3% w/v glutaraldehyde for 1 h. The fixed material was spun down at 13 000 × *g* at room temperature to form a tight pellet and resuspended in two volumes 0.1 m HEPES buffer. An aliquot (10 µl) was allowed to settle on a poly-l-lysine-coated coverslip that was rinsed in buffer to remove excess material. It was then dehydrated in ethanol and critical point-dried. A small portion of the dried material was attached to a conductive stub and sputter-coated with gold to view in the SEM.

#### Transmission electron microscopy (TEM)

Reynolds' lead citrate was prepared as previously described (Reynolds, 1963). The fixed structures, prepared as above, were washed three times in water and resuspended in a 1% aqueous solution of sodium metaperiodate for cell wall permeabilization. Free aldehyde was quenched by incubation of 5 mm ammonium chloride. The pellet was dehydrated in ethanol and infiltrated with, and embedded in, white resin in a gelatin capsule. The pellet was hardened by incubation at 45°C and sectioned at 80–120 nm thickness to stain with 2% v/v uranyl acetate and Reynolds' lead citrate for TEM.

### Density gradient fractionation using iodixanol

#### Single-step density gradient

The cell lysate was layered on top of a 14.5% v/v iodixanol (Opti-prep, Sigma) gradient and centrifuged at 28 000 rpm (96 300 × *g*) at 4°C for 18 h in a SW32 rotor (Beckman Coulter). The gradient was collected to 0.5 ml fractions on ice, using an Auto Densi-flow device (Labconco).

#### Two-step density gradient

The cell homogenate was subjected to centrifugation at 10 000 × *g* for 15 min at 4°C. The pellet was resuspended in 2 ml 250 mm sucrose, 10 mm HEPES (pH 7.4) and 1 mm EDTA (pH 8.0) buffer, and layered on top of a 10 mm HEPES-buffered sucrose step gradient (2 ml, 15%; 3 ml, 25%; 3 ml, 40%; and 2 ml, 60%, respectively) and centrifuged for 1 h at 4°C at 32 000 rpm in a SW32Ti rotor (Beckman). A reddish-brown mitochondria-enriched layer was visible after equilibration and was collected into a 2 ml centrifuge tube. The supernatant was mixed with 50% v/v iodixanol, 250 mm sucrose, 10 mm HEPES (pH 7.4) and 1 mm EDTA buffer and subjected to an 18 h centrifugation at 96 300 × *g* at 4°C in a SW32Ti rotor.

### TCA/acetone precipitation

Fractions of subcellular compartments were mixed at a ratio of 1:8:1 of fraction:100% ice-cold acetone:100% trichloroacetic acid and allowed to precipitate at −20°C overnight with gentle rocking. Proteins were recovered by centrifugation at 13 200 × *g* for 15 min at 4°C in a benchtop centrifuge. After the supernatant was discarded, the pellet was washed three times with 1 ml ice-cold acetone and dried in a nitrogen flow.

### Protein quantification and immunoblotting

Protein concentrations were determined using the BCA protein assay kit (Thermo Scientific). The subcellular compartment distribution after fractionation was analysed by western blotting, using antibodies raised against organelle marker proteins: Vps10p for Golgi (Cooper and Stevens, 1996); Dpm1p for ER (Faulhammer *et al*., 2004); Pma1p for plasma membrane (Bagnat *et al*., 2001); porin for mitochondria (Mihara and Sato, 1985), using the manufacturer's recommended protocols for the iBlot system (Invitrogen, 2011). Densitometry of visual results was quantified using ImageJ v. 1.47 (Lind, 2012).

For a detailed, stepwise protocol for yeast subcellular fractionation, see supporting information.

## Results and discussion

**Figure 1:**
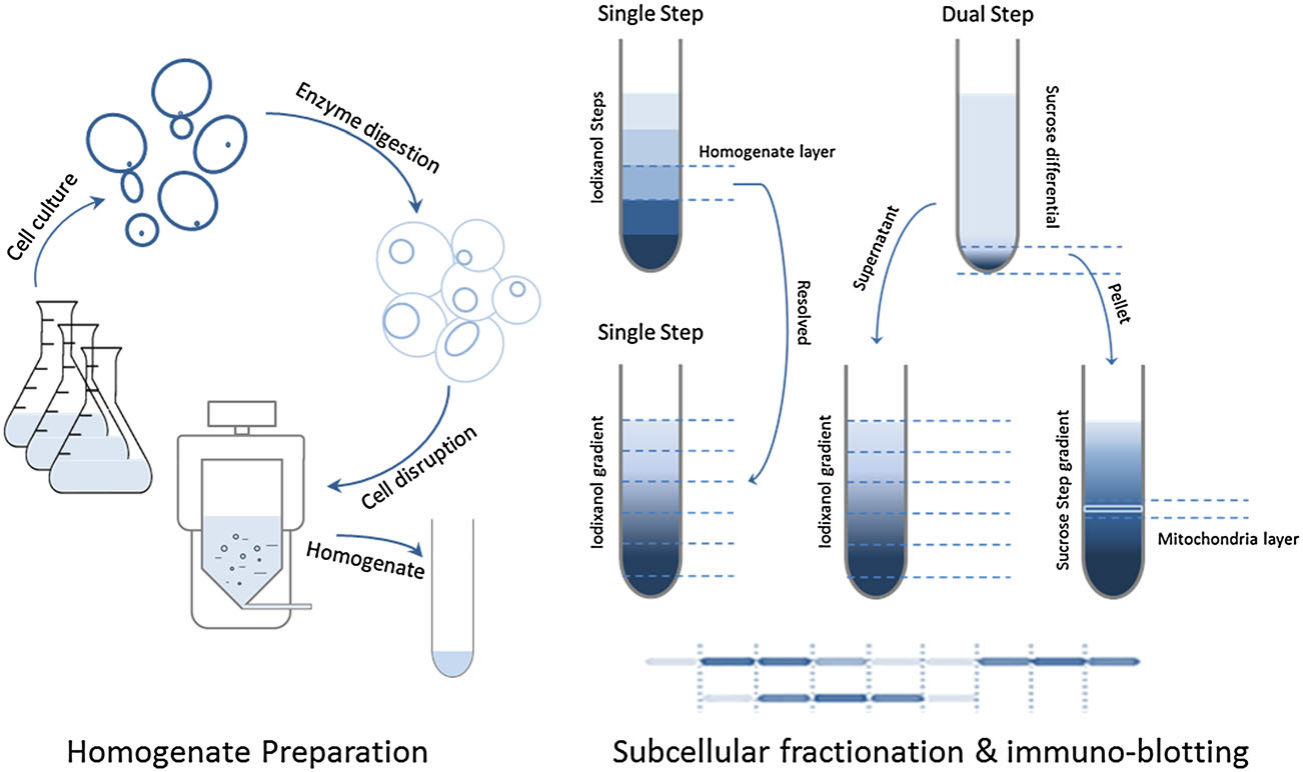
The general scheme of our protocol

Figure 1 illustrates the general scheme of our protocol; it comprises cell culture, spheroplasting, homogenization and subcellular fractionation. Yeast cells lacking cell walls, referred to as spheroplasts, are fragile and can be lysed using far more gentle methods than can whole yeast cells. In this study, the removal of the cell wall was found to be essential for the application of nitrogen decompression homogenization. The undigested yeast cells were subjected to nitrogen decompression in a pressure vessel, which was charged to 1000 psi and then released. Electron microscopic examination of the resulting product suggested that most cells remain intact. The wall-lytic enzyme preparation, Zymolyase, was used to generate spheroplasts (see Materials and methods). However, Zymolyase contains a mixture of enzymes that may degrade proteins in addition to the mannan and glucan polysaccharides of the yeast cell wall. This could lead to the removal of physiologically important proteins, such as plasma membrane transporters. For this reason, spheroplasts must be generated in the minimum possible time and then washed immediately and extensively, to remove not only Zymolyase but also the reducing reagent DTT. Spheroplasts are very sensitive to mechanical and osmotic stress and must be suspended in an isotonic solution during and after the digestion procedure to prevent lysis. In this study, the non-metabolizable sugar, sorbitol, was used at a concentration of 1.0 m to provide osmotic support.

The major desiderata for a homogenization technique are that it should break a high proportion of the spheroplasts while causing minimal damage to the subcellular compartments, and show a high degree of reproducibility. Current spheroplast lysis methods can be divided into those applying mechanical shear forces under isotonic conditions, and those that involve a rapid reduction in osmotic support. Although osmotic shock is a gentle and controllable method (Bauer *et al*., 1979; Hupfeld *et al*., 2010), the released organelles may also be perturbed. While the widely practised mechanical lysis methods provide rapid and efficient homogenization, they exhibit poor reproducibility and often cause extensive damage to organelle integrity, with accompanying loss of associated macromolecules (Carbonero *et al*., 2011).

**Figure 2:**
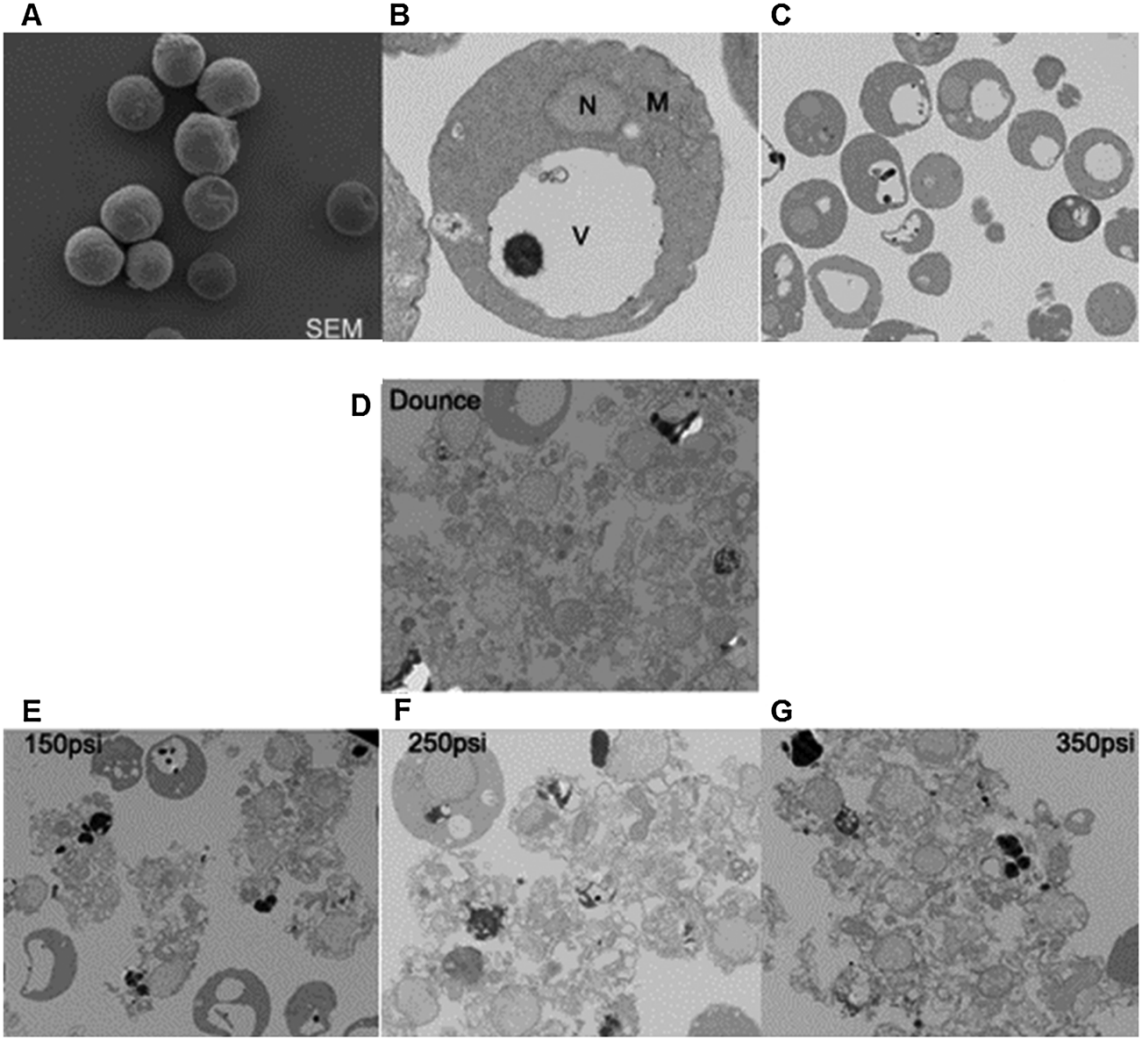
Ultrastructure of spheroplasts and their lysates. (A) SEM of intact spheroplasts, bud scars and wrinkles on the surface were observed. (B, C) TEM of intact spheroplasts: V, vacuole containing polyphosphate; N, nucleus; M, mitochondrion. (D) Cell homogenate generated by the Dounce procedure. (E–G) Cell homogenates generated by nitrogen cavitation at 150, 250 and 350 psi

**Figure 3:**
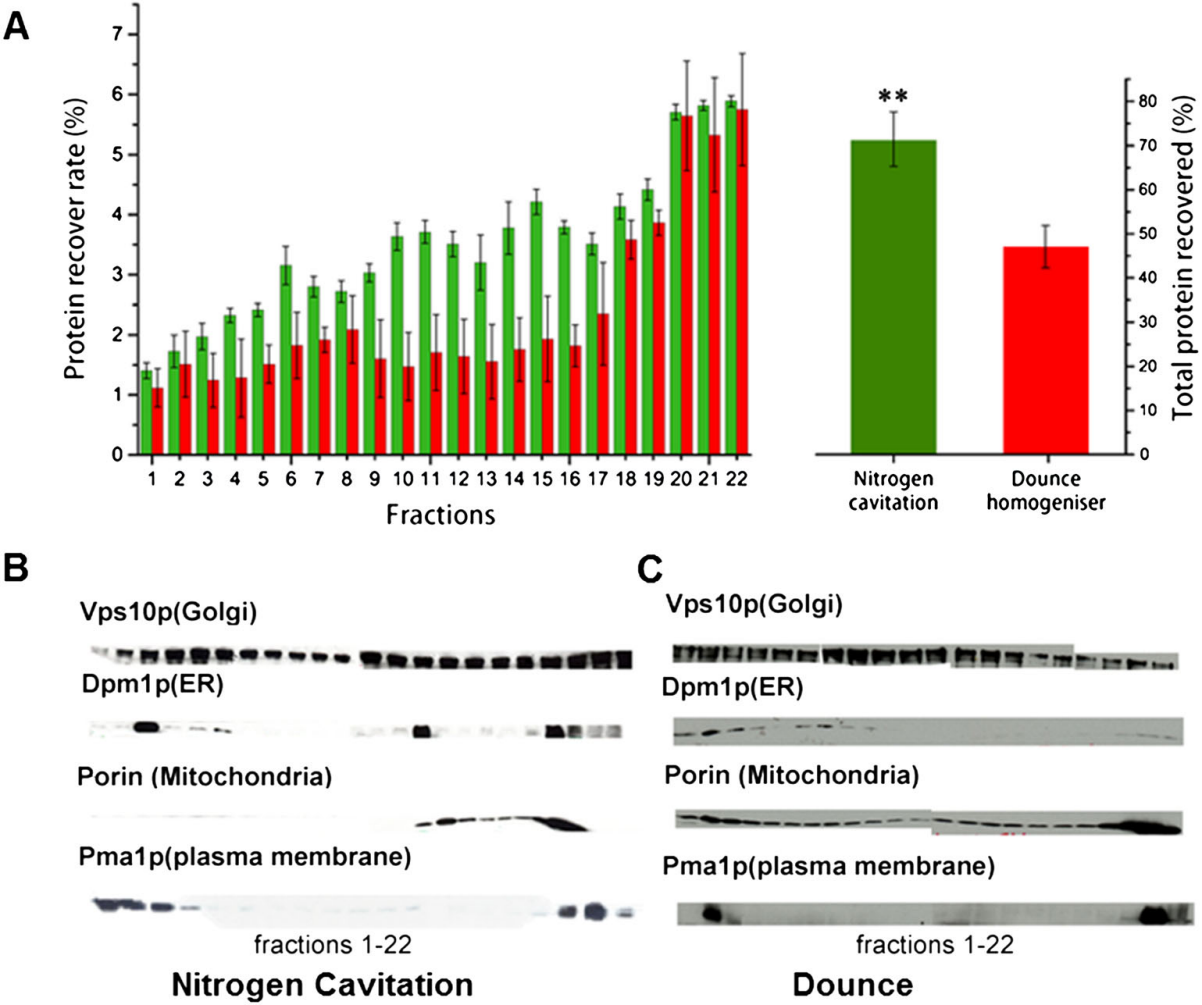
Performance of the Dounce and nitrogen cavitation methods compared in triplicate experiments using identical density gradients. (A) Comparison of protein recovery rate of Dounce homogenization (red) and nitrogen cavitation (green) in each fraction: (left panel) percentage of total cell protein recovered in each fraction; (right panel) percentage of total cell protein recovered for the whole gradient; *p < 0.05; **p < 0.005. (B) Representative western blotting data, showing organelle resolution in a 14.5% v/v iodixanol density gradient loaded with homogenate, prepared by nitrogen cavitation. (C) Representative western blotting data, showing organelle resolution in a 14.5% v/v iodixanol density gradient loaded with homogenate, prepared by Dounce homogenization

In this study, the nitrogen decompression and mechanical lysis methods were initially compared using electron microscopy (Figure 2). As might be expected, the higher the initial pressure applied to the spheroplasts, the more vigorous the cell disruption effected by nitrogen decompression. Since, in nitrogen cavitation, the same disruptive forces are applied within each cell and throughout the sample (Simpson, 2010), incomplete or excessive breakage of cells can be mitigated by adjusting the nitrogen pressure to achieve efficient organelle release from cells. In a comparison of protein recoveries, using an identical preliminary density gradient, nitrogen cavitation demonstrated significantly better performance than Dounce homogenization (Figure 3A), protein recovery being 46% higher. Moreover, a comparison of iodixanol gradient fractions, using immunoblotting with antibodies against organelle marker proteins, demonstrated that nitrogen cavitation permitted better resolution between the major intracellular organelles than did Dounce homogenization. This indicated that nitrogen cavitation was better at maintaining the integrity of subcellular compartments (cf. Figure 3B, C).

**Figure 4:**
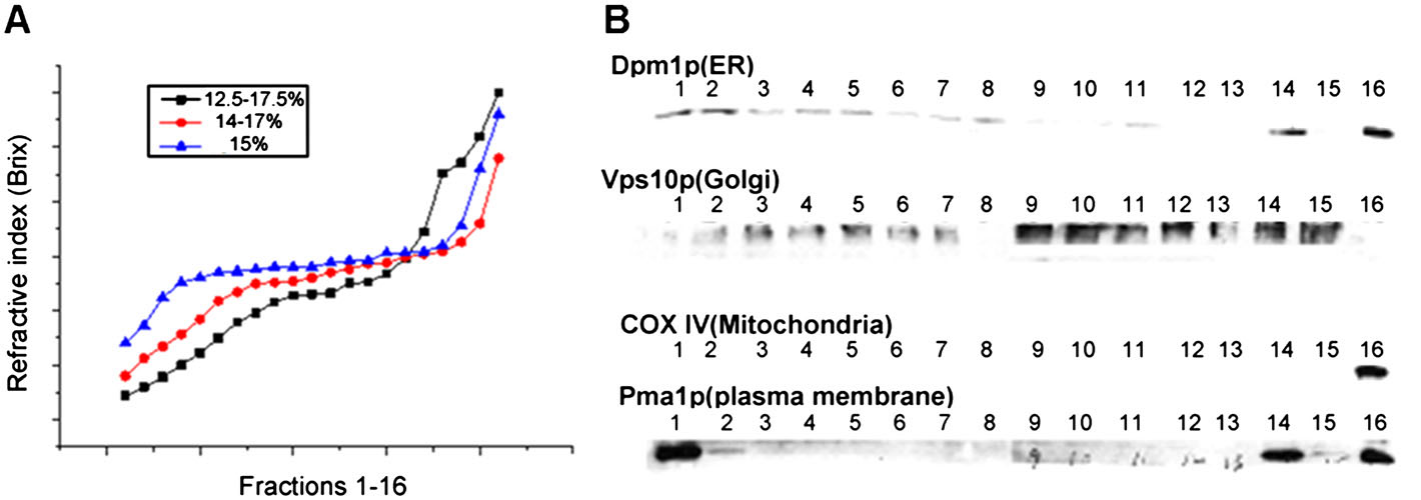
(A) Density gradient of fractions form buoyant density centrifugation without an iodixanol cushion for membrane enrichment. (B) Representative western blotting data, showing organelle resolution in the 14–17% single gradient that was selected for maximum organelle resolution

**Figure 5:**
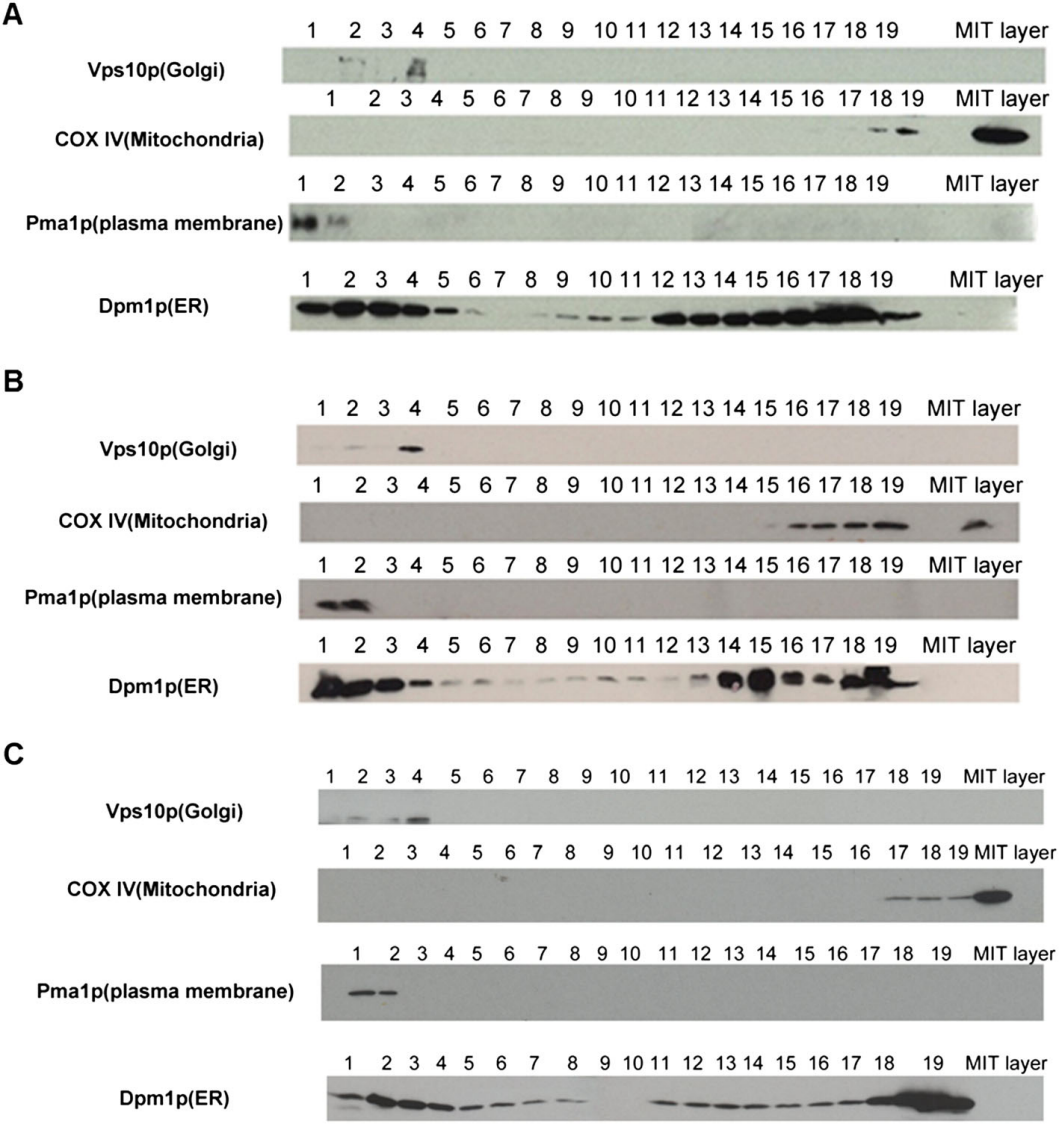
Western blot against organelle marker proteins of yeast cell lysates, fractionated using a dual gradient, MIT layer on the right side is the separated mitochondrial layer of a sucrose step gradient; 1–19 fractions were collected from an iodixanol continuous gradient. Three biological replicates (A–C) were performed, using the same protocol; the resulting fractionation of yeast organelles shows a very similar distribution profile in the three replicates

Iodixanol itself generates a density gradient upon centrifugation. The profile of the gradient generated depends on the applied centrifugal force, centrifugation time and iodixanol concentration. Various gradient profiles were compared in order to optimize subcellular fractionation. A shallower middle region was observed when using multiple-density layered iodixanol gradients, where Golgi apparatus and ER showed partial separation instead of overlapping enrichment (Figure 4A; see also supporting information, Figure S1). Although a precentrifugation using a cushion of high-percentage iodixanol is considered beneficial to concentrate the subcellular compartments (Hagen, 2008), such a procedure may also perturb organelle integrity and thus compromise the separation performance. A single gradient without prior iodixanol cushioning was chosen for maximal organelle resolution (Figure 4). However, the resolution of the endomembrane system from single-gradient separation was still limited by the poor separation of the denser organelles from the cell debris. In addition, there was inadequate resolution of the endomembrane system from the mitochondria. In order to solve both these problems, a two-step separation, comprising a sucrose step gradient for isolation of mitochondria and an iodixanol continuous gradient for the less dense organelles, was developed. As Figure 5A demonstrates, the mitochondria are highly enriched in one sucrose density layer and their integrity was validated by western blotting, using an antibody against cytochrome *c* oxidase subunit IV (COX IV), which is the terminal member of the mitochondrial inner membrane electron transport chain (Cooper *et al*., 1991).

**Figure 6:**
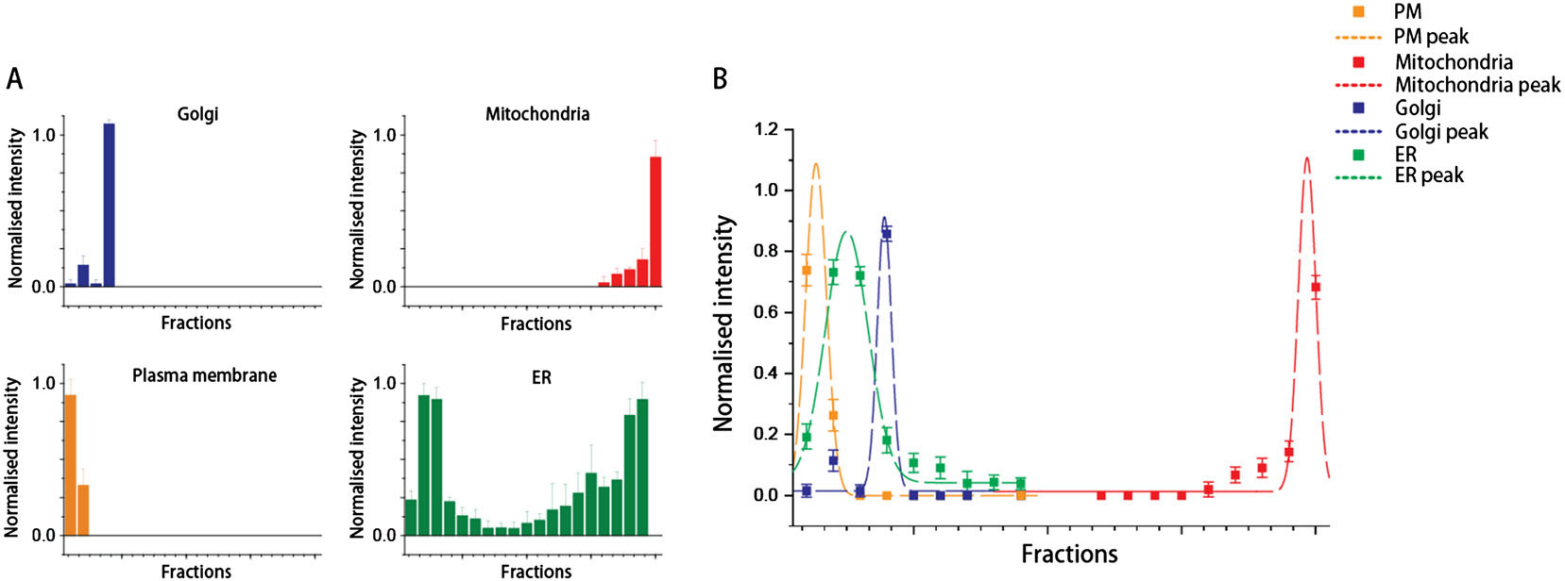
Quantitative analysis of immunoblotting signals. (A) Average signal intensities for organelle markers are normalized and plotted across the entire gradient. Error bars (standard deviation, SD) indicate that the majority of fractions (even those with relatively low protein concentrations) showed a high level of reproducibility. (B) Gaussian peak fit for organelle-enriched fractions. Gaussian functions were fitted to the original data (dots with relative SDs) by using a damped least-squares (Levenberg–Marquadt) algorithm. The resulting Gaussian peaks were validated by reduced *χ*^2^ value, residual sum of squares and *F* value. Plasma membrane (PM), ER, Golgi and mitochondria reporters showed peaks at distinctive fractions, confirming the high degree of resolution achieved by this protocol

The optimized protocol was performed three times under the same conditions and the results were compared (Figure 5). Despite differences in scale between the different repeat experiments, the fractionated organelles demonstrate similar distribution profiles (Figure 6A), with major organelles resolved (Figure 6B), demonstrating that the protocol is highly reproducible.

## Conclusion

Many protocols have been developed to separate organelles in yeast, either for comprehensive analyses or to investigate specific subcompartments. The protocol described here can be used to obtain highly enriched ER, Golgi, plasma membrane and mitochondria fractions simultaneously and, if properly modified, can also be used to isolate the major organelles from other yeast species protected by a tough cell wall. In comparison with direct mechanical disruption, enzymatic digestion methods tend to expose cells to an extensive period of stress prior to lysis, and this can conflict with the goals of some experiments. On the other hand, such treatments can reduce the disruptive force required to lyse the cells, and thus mitigate the potential damage to both organelles and large protein complexes. An alternative cell lysis method to mechanical homogenization, that of osmotic shock, is usually considered to be a more gentle approach to releasing organelles. However, it may cause even more disruption to internal organelles than to the spheroplasts themselves, thus compromising the goal of simultaneously achieving the high-resolution separation of all subcellular organelles and membrane classes. The nitrogen cavitation method avoids many of these problems. Moreover,we have obviated the need for frequent transfers between different buffer environments during the homogenization procedure, which has also permitted an integrated centrifugation protocol that resolves all classes of organelles and membranes. Our protocol has established a facile, robust and highly reproducible approach that can be used in the investigation of the impact of compartmentalization on metabolic processes and macromolecular interactions within the yeast cell. It is hoped that this will enable quantitative studies to be carried out that will allow the accurate representation and integration of subcellular compartmentation in systems biology models.

## Supporting Information

Figure S1 Western blot of iodixanol density grandient (15% and 12.5–17.5%) fractions against organelle markers Dpmlp (ER) and Vps10p (Golgi). The difference between ER and Golgi are insignificantly distinctive, thus these two gradients were not considered.
