# Supplementary figures and images for: A protocol for the subcellular fractionation of Saccharomyces cerevisiae using nitrogen cavitation and density gradient centrifugation
Source: Yeast. 2014 Feb 20;31(4):127–35. doi: 10.1002/yea.3002 (PMC4282465; doi:10.1002/yea.3002)

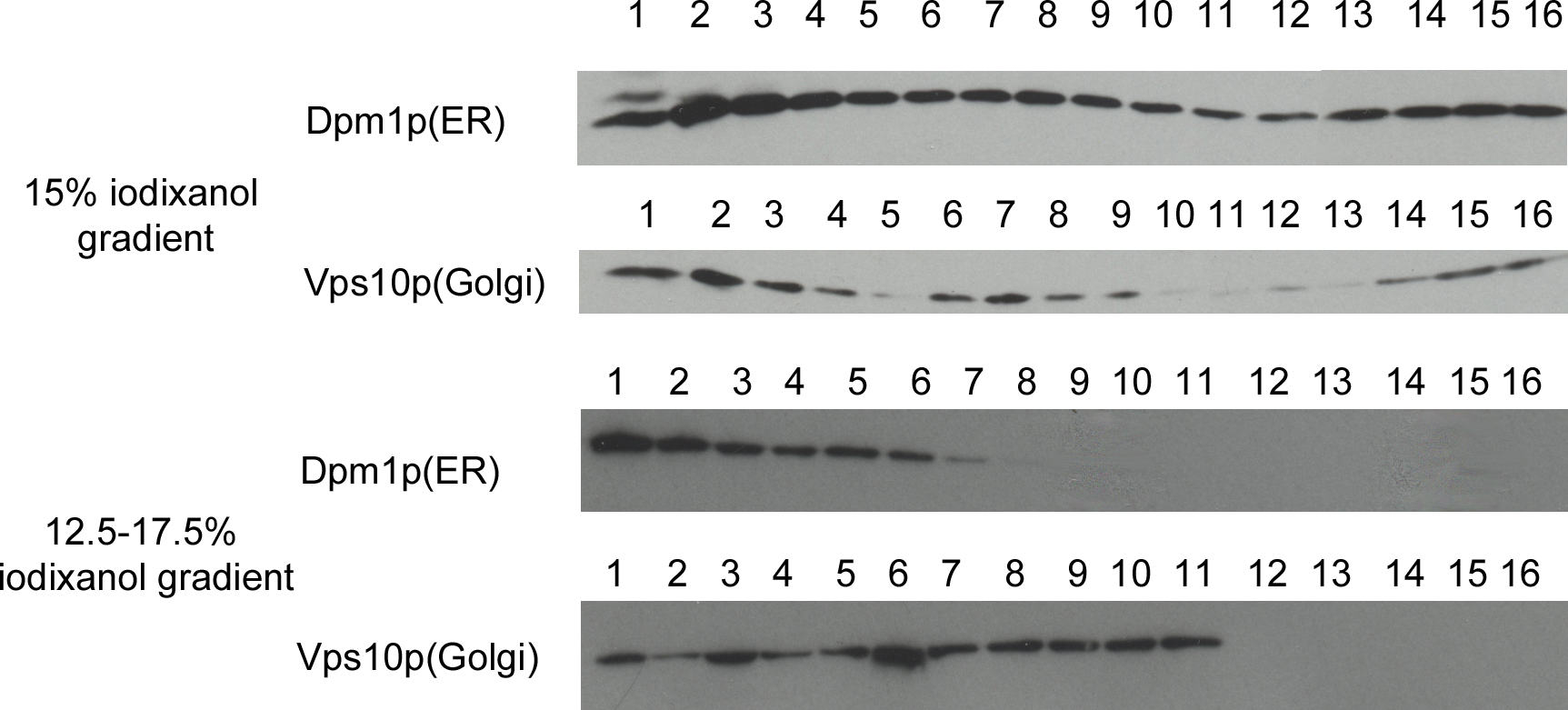

Supplement: Figure S1 — Western blot of iodixanol density grandient (15% and 12.5–17.5%) fractions against organelle markers Dpmlp (ER) and Vps10p (Golgi). The difference between ER and Golgi are insignificantly distinctive, thus these two gradients were not considered. [file yea0031-0127-SD1.tif]
