# Supplementary material for: A protocol for the subcellular fractionation of Saccharomyces cerevisiae using nitrogen cavitation and density gradient centrifugation
Source: Yeast. 2014 Feb 20;31(4):127–35. doi: 10.1002/yea.3002 (PMC4282465; doi:10.1002/yea.3002)
Supplement: Figure S1 — Western blot of iodixanol density grandient (15% and 12.5–17.5%) fractions against organelle markers Dpmlp (ER) and Vps10p (Golgi). The difference between ER and Golgi are insignificantly distinctive, thus these two gradients were not considered. [file yea0031-0127-SD2.doc]

**Supplementary information**

**Supplementary Figure 1**

Western blot of iodixanol density gradient (15% and 12.5-17.5%) fractions against organelle markers Dpm1p(ER) and Vps10p(Golgi). The difference between ER and Golgi are insignificantly distinctive thus these two gradients were not considered.

**Detailed protocol for yeast subcellular fractionation:**

**Reagents:**

D-Sorbitol (Sigma-Aldrich), DL-Dithiothreitol (Sigma-Aldrich), Sucrose (Sigma-Aldrich), Disodium EDTA (EDTA; Sigma-Aldrich), Bis-Tris (Sigma-Aldrich), Sulfuric acid solution (Sigma-Aldrich), Glycine (Sigma-Aldrich), BSA (Sigma-Aldrich), HEPES (Sigma-Aldrich), Potassium hydroxide (KOH; Sigma-Aldrich), Sodium hydroxide (NaOH; Sigma-Aldrich), OptiPrep™ Density Gradient Medium (Sigma-Aldrich), TWEEN^®^20 (Sigma-Aldrich), Dithiothreitol (Sigma-Aldrich), cOmplete Protease inhibitor (Roche), Yeast nitrogen base (YNB; Difco), Bidistilled water.

**Equipment**

Tightly fitting glass/glass Dounce homogenizer

Cell disruption bomb 4635 (Parr Instrument)

RF10 portable sucrose brix refractometer (Extech)

Ultraclear 14-ml polypropylene tubes for SW41 rotor (Beckman Coulter)

Eppendorf low-binding microcentrifuge test tubes, 1.5 ml (Eppendorf)

Refrigerated low-speed centrifuge, tabletop, for 30- or 1.5-ml tubes (Eppendorf)

Optima L-100 XP ultracentrifuge (Beckman Coulter)

SW 32 Ti Rotor, swinging bucket (Beckman Coulter)

Auto Densi-flow pump (Labconco)

**Reagent setup**

Culture medium: 20 g/L peptone, 10 g/L yeast extract, autoclave, cool to 55°C and then add sterile dextrose (glucose) to 2% w/v(YPD).

Spheroplast medium (SM): 1Xyeast nitrogen base, 2% w/v dextrose, 1M sorbital, 20mM Tris·Cl, pH 7.5. Medium was sterilised by filtering through 0.22μm filter. Supplement with protease inhibitor prior to use.

Homogenisation medium (HM): 250 mM sucrose, 10 mM HEPES (pH 7.4; buffered by KOH), 1 mM EDTA, (pH 8.0) and 1 mM DTT. Medium needs to be prepared fresh (to prevent oxidation of DTT), supplemented with protease inhibitor and prechilled to 4°C.

Working solution buffer (WSB): 1.5 M sucrose, 60 mM HEPES (pH 7.4; buffered by KOH), 6 mM EDTA, (pH 8.0) and 6 mM DTT. Buffer needs to be prepared fresh and chilled to 4°C.

Iodixanol working solution (IWS): Mix 60% OptiPrep stock solution and WSB in a 5:1 volume ratio. Solution needs to be prepared fresh, supplemented with protease inhibitor and prechilled to 4°C.

Iodixanol cushion solution: Make a cushion of the desired iodixanol concentration. For example, for an 18% (25.6 Brix) iodixanol cushion, mix 14.4 ml of IWS with 25.6ml of HM. Measure the iodixanol concentration using a refractometer and the refractive index table of iodixanol-sucrose gradient solutions available from Axis Shield. Use IWS and HM media to increase or decrease the refractive index, respectively. This cushion partially removes high-density organelles such as mitochondria and nuclei, enriching the crude membranes harvested from the interphase with the organelles of interest.

Iodixanol gradient solution: Choose the percentage of the gradient want to use. For example, mix 16.4 ml of IWS and 13.6 ml of HM for a 19% (27 Brix) gradient. Adjust the refractive index with IWS or HM. This gradient enables major organelles, e.g. the Golgi apparatus, ER and plasma membrane to be resolved.

Sucrose step gradient: dissolve sucrose in HM at 15%, 25%, 40% and 60% w/v respectively. Solutions need to be prepared fresh and prechilled to 4°C

**Procedure:**

**Pre-culture cells**

1. Start a pre-culture using *Saccharomyces cerevisiae* strain BY4743-Y23925 (*MAT****a***/*MATα his3∆1*/*his3∆1* *leu2∆0*/*leu2∆0* *ho*::kanMX/*ho*::kanMX), by inoculating 20mL YPD with a single yeast colony from a fresh YPD agar plate.

**Cell culture**

1. Allow pre-culture to reach late-exponential phase before measuring the cell density. Inoculate 200mL YPD medium with pre-cultured cells to a final cell density of OD*_600_* = 0.001. Culture cells whilst shaking at 250rpm at 30°C for ~18hrs to reach mid-exponential phase.

**Spheroplast preparation**

1. Measure the density of cell culture. Sample a volume containing 200 OD_600_ units of yeast cells.
2. Harvest cells by 3000×g centrifugation from mid-exponential phase culture at room temperature, and discard supernatant.
3. Resuspend cells at 20mL (10 OD_600_ units/mL) in 100mM Tris-sulphate buffer (pH 9.4) supplemented with 10mM dithiothreitol, for 10min.
4. Harvest cells by centrifuging 10min at 3000×g. Aspirate supernatant and Resuspend cells in 10mL (20 OD_600_ units/mL) in SM at room temperature.
5. Add 200μg Zymolase 100T to the medium (1μg/OD_600_ unit), shaking at 30°C for 10min.
6. Harvest spheroplasts by centrifuging 5min at 1500×g, room temperature. Resuspend the pellet in HM thoroughly to wash spheroplasts twice. Harvest by centrifuging 5min at 1500xg, 4°C.

Lyse spheroplasts using nitrogen cavitation

1. Resuspend the spheroplasts in 20mL (10 OD_600_ units/mL) in HM and transfer to a pre-chilled cell disruption bomb.
2. Connect the bomb to a nitrogen source and charge it to 500psi on ice for 10min. Then release the pressure to 350psi from inlet valve. Leave on ice for 5min.
3. Collect cell homogenate from outlet valve to a 50mL sterile polypropylene centrifugation tube. Dilute homogenate using equal volume of HM.
4. Clear unbroken cells, partially disrupted cells, and aggregates by centrifuging 5min at 1000×g, 4°C.
5. Discard the pellet and centrifuge the supernatant again for 5 min at 3000×g, 4°C
6. Discard the pellet (if present) and centrifuge the supernatant for 10 min at 10,000×g, 4 °C.
7. Collect both the pellet as crude mitochondrial fraction and supernatant as mixture of other lighter organelles. To process the pellet, continue with Step 16-20. To process the supernatant, continue with Step 21-24.

**Sucrose step gradient to purify mitochondria**

1. Resuspend the mitochondrial pellet from Step.15 thoroughly in 10 mL ice-cold HM and centrifuge for 10 min at 8,000×g, 4 °C.
2. Aspirate supernatant and resuspend pellet in 2mL (100 OD_600_ unites/mL) HM.
3. Lay a step sucrose gradient in an ultra-centrifugation polypropylene tube using 2mL 15%, 3mL 25%, 3mL 40% and 2mL 60% sucrose gradient solutions respectively.
4. Carefully lay the crude mitochondrial fraction on top of the sucrose step gradient.
5. Centrifuge the tube for 1 hour at 32000rpm (125,588×g), 4°C in a SW32Ti rotor. A reddish-brown mitochondria-enriched layer will visible after equilibration and can be collected into a 2mL centrifuge tube.

**Iodixanol continuous gradient to resolve lighter organelles**

1. Centrifuge the supernatant from Step.15 for 10 min at 10,000×g, 4 °C. Collect supernatant.
2. Adjust the iodixanol concentration to 14.5%v/v (24.0 brix) by mixing 14.2mL of resulting supernatant with ~5.8mL ice cold IWS. Transfer the medium to an ultra-centrifugation polypropylene tube.
3. Centrifuge the tube for 18hours at 28,000rpm (96,300×g), 4°C using a SW32Ti rotor in a Beckman Coulter Optima L-100 XP ultracentrifuge (accel = 9, decal =6).
4. Collect the gradient into 20 fractions using density-flow pump into 1.5mL low protein-binding tubes.

Fractions containing separated organelles should be frozen at −80 °C for up to 6 months or at −20 °C for up to 1 month if it is not used immediately.
